# Supplementary material for: Elevated glutamate impedes anti-HIV-1 CD8 + T cell responses in HIV-1-infected individuals on antiretroviral therapy
Source: Commun Biol. 2023 Jul 7;6:696. doi: 10.1038/s42003-023-04975-z (PMC10328948; doi:10.1038/s42003-023-04975-z)
Supplement: Supplementary file 2 — Supplementary Information [file 42003_2023_4975_MOESM2_ESM.pdf]

Supplementary information for

Elevated glutamate impedes anti-HIV-1 CD8+ T cell responses in HIV-1-infected individuals on antiretroviral therapy

Supplementary Fig 1. Gender differences exist in the correlation between plasma glutamate and HIV-1 reservoir.

Supplementary Fig 2. Glutamate positively correlated with CCL4+ CD8+ T cells or TVM cells.

Supplementary Fig 3. Single-cell transcriptional analysis of CD8+ T cells.

Supplementary Fig 4. Representative flow cytometry plots and expression characteristics of *EOMES*.
Supplementary Fig 5. Single-cell transcriptional analysis of human TVM cells. **Supplementary Figure 6.** Single-cell transcriptional analysis for HC, TN and ART group.

Supplementary Fig 7. Glutamate inhibited CD8+ T and TVM cell function ex vivo.

Supplementary Fig 8. Glutamate affects TVM cell function in treatment naive PLWH.

Supplementary Fig 9. The impacts of glutamate metabolism on the anti-HIV function of TVM cells.

Supplementary Table 1. Clinical characteristics of participants enrolled in plasma metabolites detections.
Supplementary Table 2. Comparison of glutamate levels between different clinical groups.
Supplementary Table 3. Signature genes usded to calculate of functional scores.
Supplementary Table 4. Mass spectrum Multiple Reaction Monitoring acquisition parameters.

Figure S1

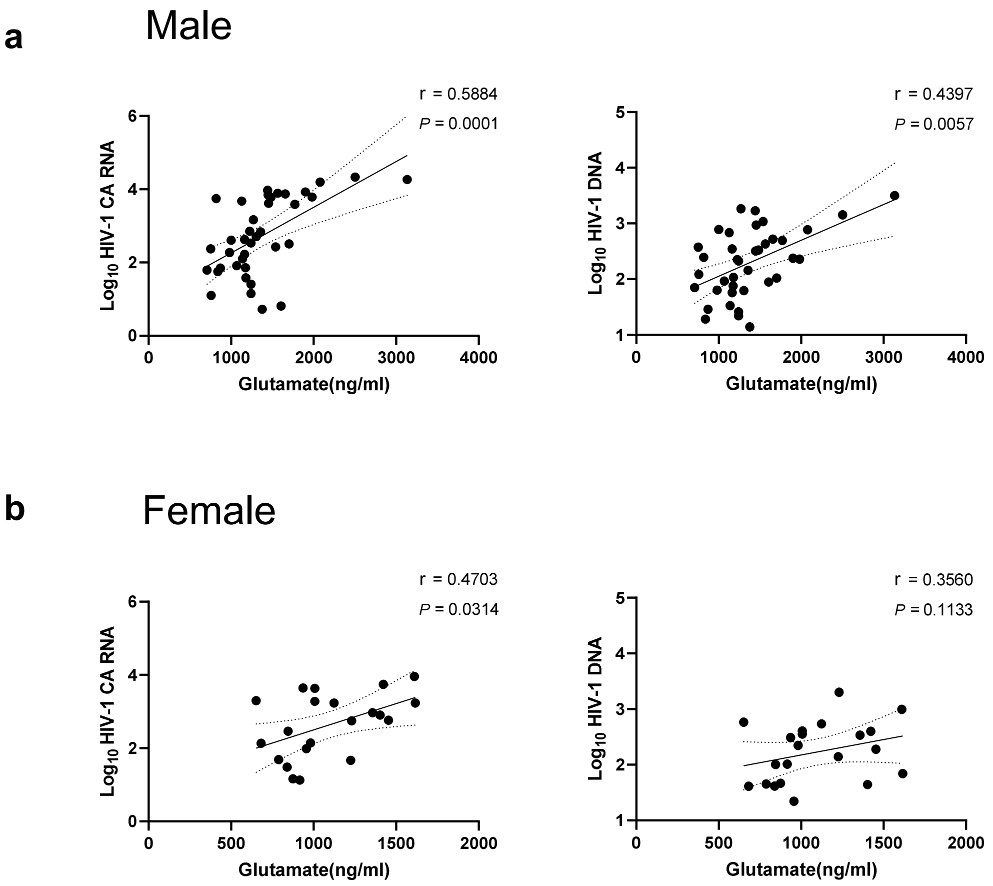

Supplementary Fig 1. Gender differences exist in the correlation between plasma glutamate and HIV-1 reservoir. **a** Correlation between plasma glutamate and HIV-1 DNA or CA usRNA levels in male PLWH (n=38). **b** Correlation between plasma glutamate and HIV-1 DNA or CA usRNA levels in female PLWH (n=21). The correlations were evaluated using nonparametric Spearman correlation tests. Nonparametric Spearman's r and p values are presented.

Figure S2

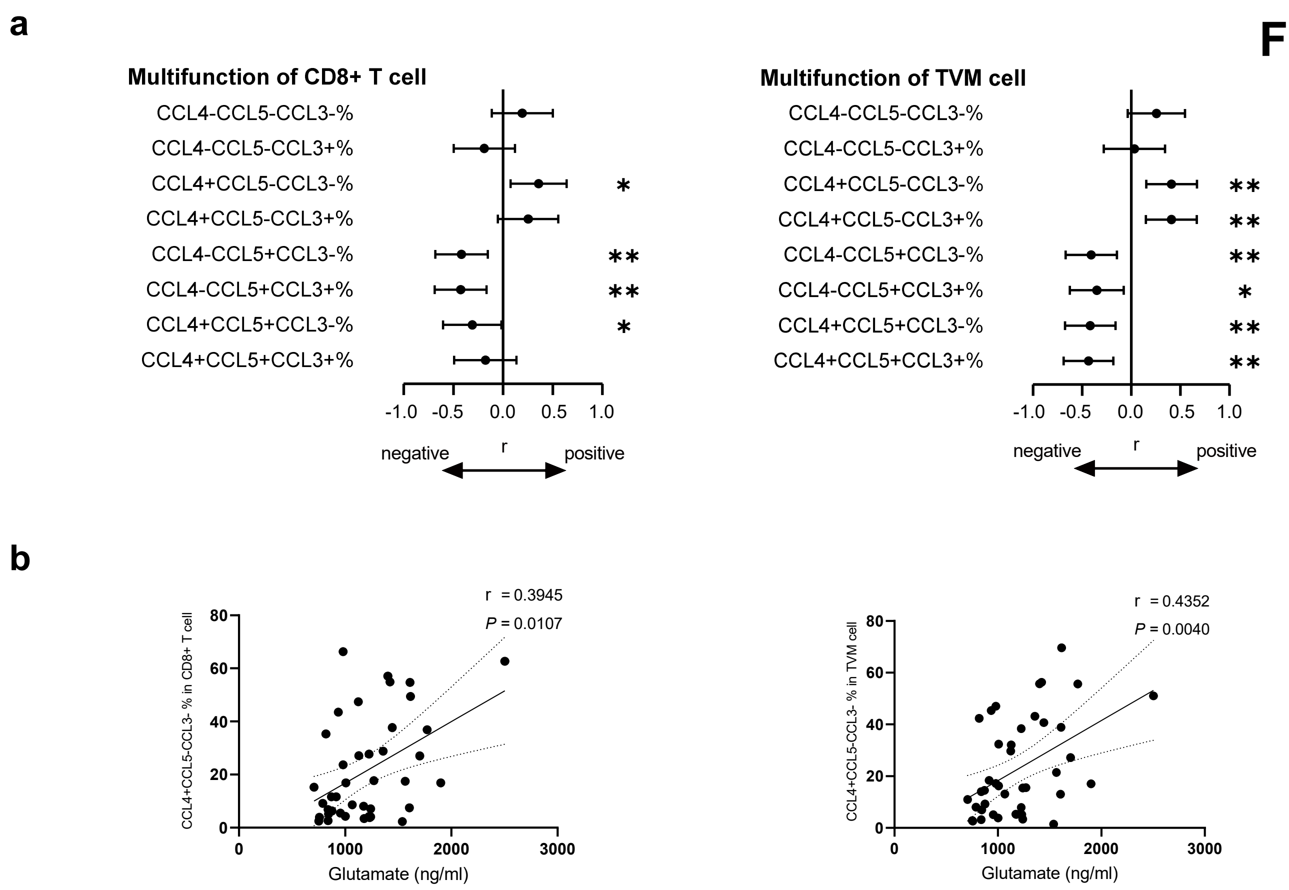

Supplementary Figure 2. Glutamate positively correlated with CCL4+ CD8+ T cells or TVM cells. **a** Correlation of plasma glutamate and poly-functional CD8+ T cell and TVM cell percentages with CCL3, CCL4, CCL5 secretion. **b** Correlation of plasma glutamate and CCL4+CCL5-CCL3-% in CD8+ T cell or in TVM cell. The correlations were evaluated using nonparametric Spearman correlation tests. Nonparametric Spearman's r and p values are presented. **b** Black dots denote nonparametric Spearman r, and black lines denote 95%confidence interval. *P < 0.05, **P < 0.01

Figure S3

a

Characteristics of individuals in scRNA-seq analysis

	HC				TN					ART		
Patient ID	HC01	HC02	HC03	HC04	P01	P02	P04	P07	P08	P10	P11	P12
Gender	Male	Male	Male	Male	Male	Male	Male	Male	Male	Male	Male	Male
Age (years)	27	31	27	30	26	40	43	21	25	24	29	32
ART regimen	-	-	-	-	-	-	-	-	-	3TC/TDF/EFV	3TC/AZT/NVP	3TC/TDF/EFV
Time on ART (m)	-	-	-	-	-	-	-	-	-	24	70	24
CD4+ T cell count (cells/μl)	816	525	1134	822	348	383	289	302	266	457	459	311
CD8+ T cell count (cells/μl)	634	352	940	621	1352	1659	854	1450	1049	604	618	460
CD4/CD8 ratio	1.29	1.49	1.21	1.32	0.26	0.23	0.34	0.21	0.25	0.76	0.74	0.68
Viral load (log10 copies/ml)	-	-	-	-	4.51	4.45	5.28	6.5	4.13	<LOD	<LOD	<LOD

b

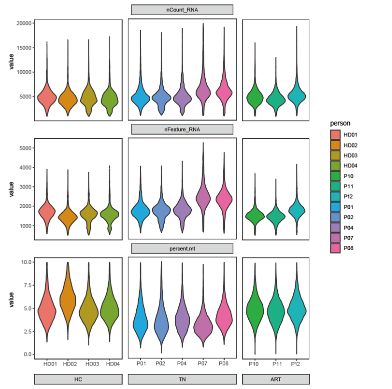

c

d

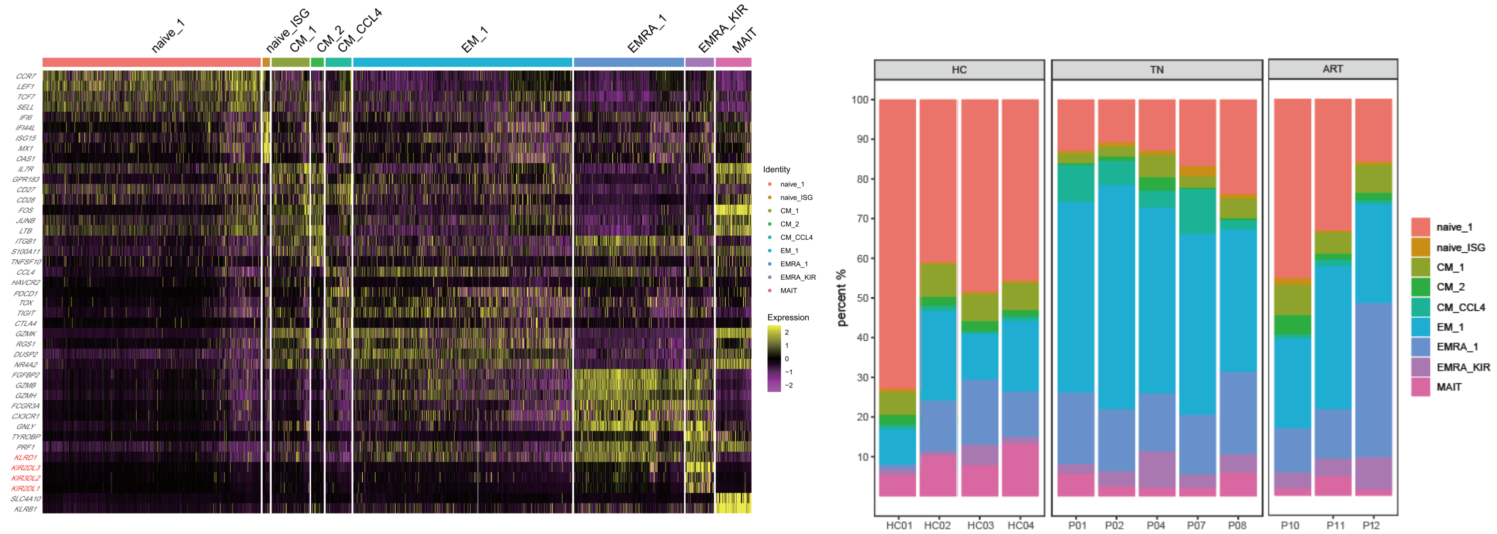

Supplementary Figure 3. Single-cell transcriptional analysis of CD8+ T cells. **a** Clinical information for individuals in scRNA-seq analysis. **b** Number of read counts (top), number of feature read counts (middle), and percentage of mitochondrial genes (down) in each sample. **c** Heat map showed the gene expression distribution of selected typical cellular markers in nine immune subpopulations of CD8+ T cells. **d** The proportion of different CD8+ T cell immune subpopulations in each individual. 3TC, Lamivudine; TDF, Tenofovir; EFV, Efavirenz; AZT, Zidovudine; NVP, Nevirapine. LOD, limit of detection

Figure S4

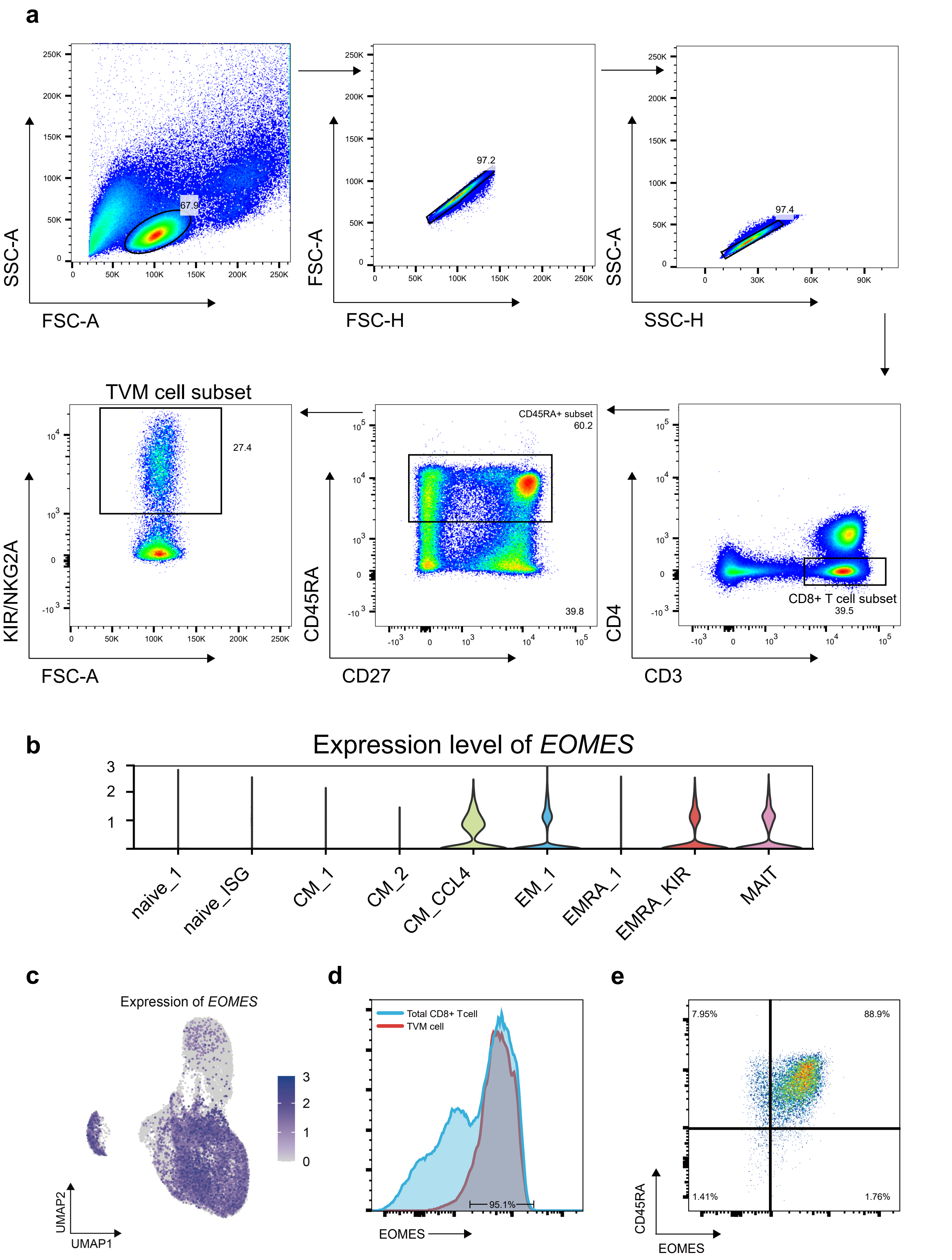

Supplementary Figure 4. Representative flow cytometry plots and expression characteristics of *EOMES*. **a** Gating strategies for the analysis of TVM cells. **b** Violin plot of EOMES expression levels in CD8+ T cell subpopulations. **c** UMAP plot showing the distribution of EOMES expression in CD8+ T cell subpopulations, and color intensity represents the level of EOMES expression. **d** Typical flow cytometry plots showing the expression characteristics of EOEMS in the total CD8+ T cell population and the TVM cell subpopulation. **e** Typical flow cytometry plots showing the expression of CD45RA and EOMES in pan-KIR/NKG2A+ CD8+ T cells.

Figure S5

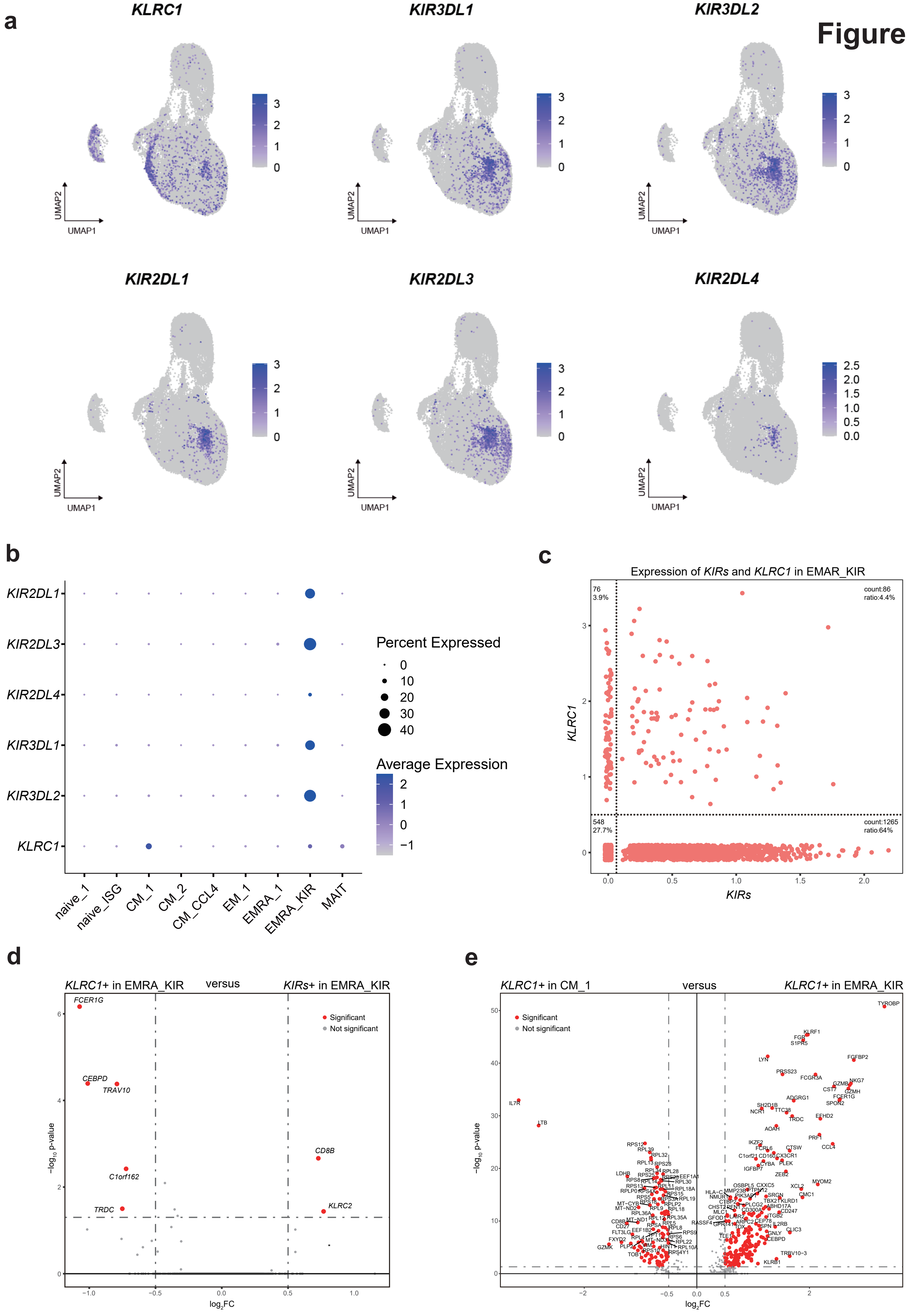
a
KLRC1
KIR3DL1
KIR3DL2
KIR2DL1
KIR2DL3
KIR2DL4
UMAP1
UMAP2
b
KIR2DL1
KIR2DL3
KIR2DL4
KIR3DL1
KIR3DL2
KLRC1
naive_1
naive_ISG
CM_1
CM_2
CM_CCL4
EM_1
EMRA_1
EMRA_KIR
MAIT
Percent Expressed
Average Expression
c
Expression of KIRs and KLRC1 in EMAR_KIR
76
3.9%
count:86
ratio:4.4%
548
27.7%
count:1265
ratio:64%
KLRC1
KIRs
d
KLRC1+ in EMRA_KIR
versus
KIRs+ in EMRA_KIR
Significant
Not significant
FCER1G
CEBPD
TRAV10
C1orf162
TRDC
CD8B
KLRC2
−log10 p-value
log2FC
e
KLRC1+ in CM_1
versus
KLRC1+ in EMRA_KIR
Significant
Not significant
TYROBP
KLRF1
FGR
S1PR5
LYN
FGFBP2
PRSS23
FCGR3A
GZMB
NKG7
CST7
GZMH
FCER1G
SPON2
ADGRG1
IL7R
LTB
SH2D1B
NCR1
TTC38
TRDC
EFHD2
AOAH
PRF1
CCL4
MYOM2
XCL2
CMC1
KLRB1
TRBV10−3
GZMK
TOB1
−log10 p-value
log2FC

Supplementary Figure 5. Single-cell transcriptional analysis of human TVM cells. **a** UMAP plot showing the distribution of *KLRC1* or *KIRs* expression in CD8+ T cell subpopulations, and color intensity represents the level of eomes expression. **b** Dot plots showing the expression of *KLRC1* and *KIRs* in the CD8+ T cell subpopulations. **c** Expression characteristics of *KLRC1* and *KIRs* in EMRA_KIR subpopulation. **d** Volcano plot showing the gene expression differences between *KIRs*+ EMRA_KIR group and *KLRC1*+ EMRA_KIR group. **e** Volcano plot showing the gene expression differences between *KLRC1*+ EMRA_KIR group and *KLRC1*+ CM_1 group.

Figure S6

a b

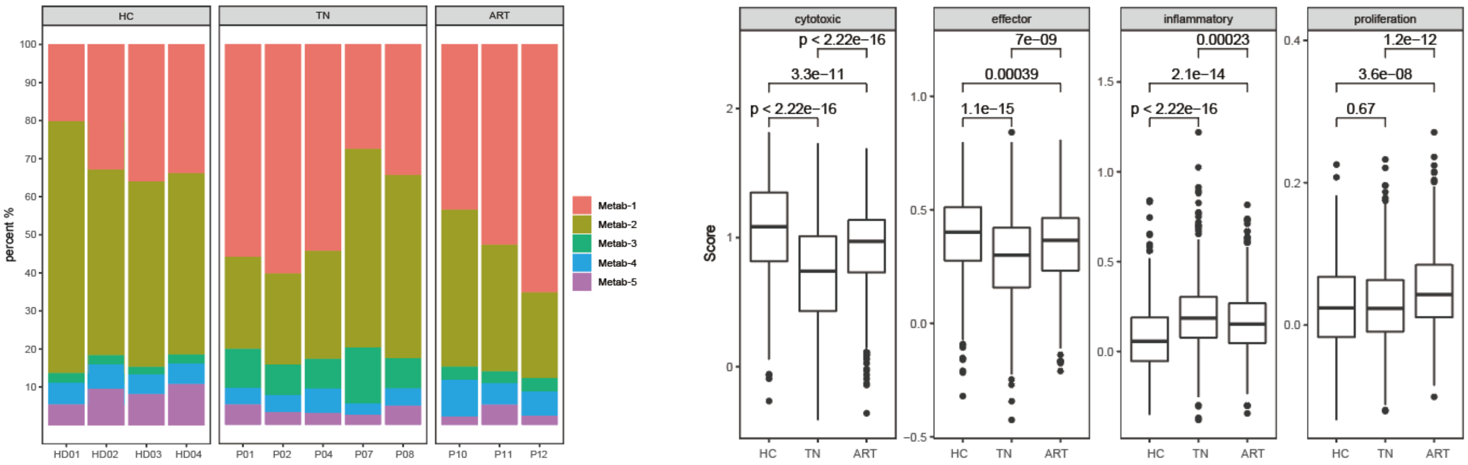

Supplementary Figure 6. Single-cell transcriptional analysis for HC, TN and ART group. **a** The proportion of different CD8+ T cell metabolic subpopulations in each individual. **b** Comparison of TVM cell function scores among TN, ART and HC groups.

Figure S7

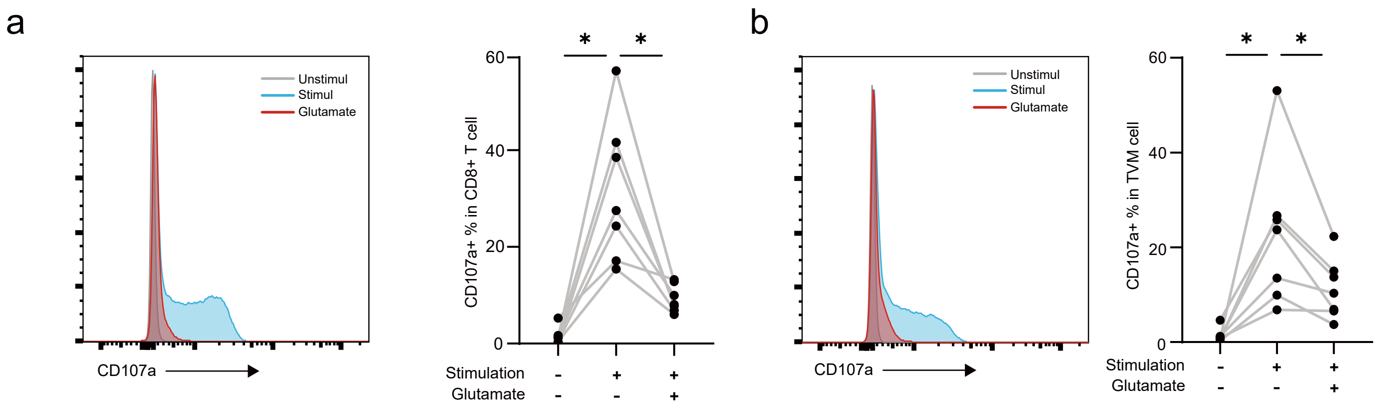

Supplementary Figure 7. Glutamate inhibited CD8+ T and TVM cell function ex vivo. **a** Left: Representative CD107a expression in CD8+ T cell of unstimulated (grey), stimulated (blue) and 5mm glutamate intervention(red). Right: Summarized data show CD107a+% in CD8+ T cells following glutamate intervention. **b** Left: Representative CD107a expression in CD8+ T cell of unstimulated (grey), stimulated (blue) and 5mm glutamate intervention(red). Right: Summarized data show CD107a+% in CD8+ T cells following glutamate intervention.

Figure S8

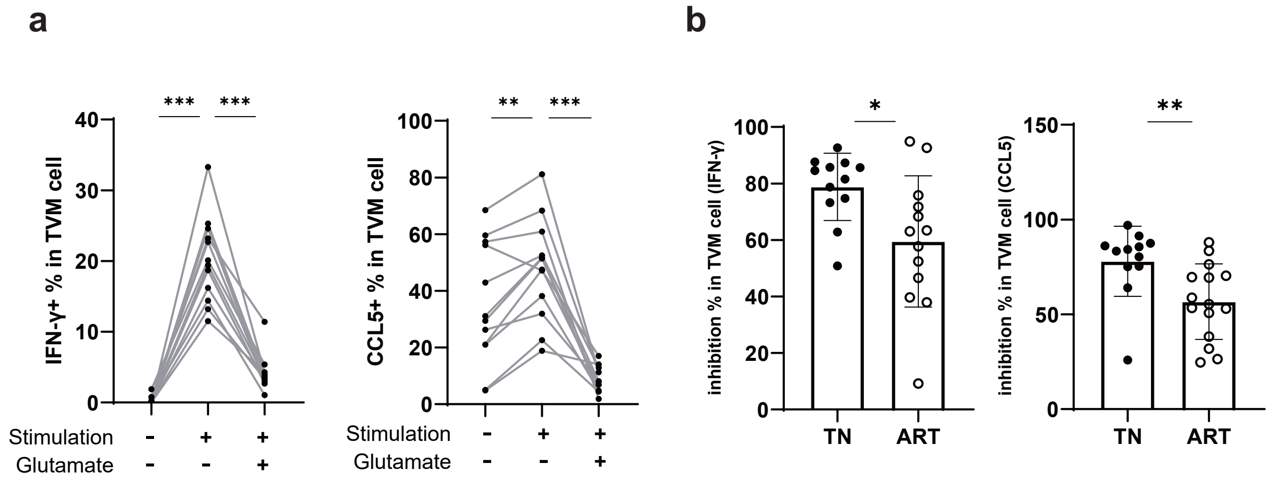

Supplementary Figure 8. Glutamate affects TVM cell function in treatment naive PLWH. **a** The effect of glutamate on TVM cell anti-HIV functions in TN group. **b** Comparison of the inhibitory rate of glutamate on the anti-HIV function of TVM cells between ART and TN groups. *P < 0.05, **P < 0.01, ***P < 0.001

Figure S9

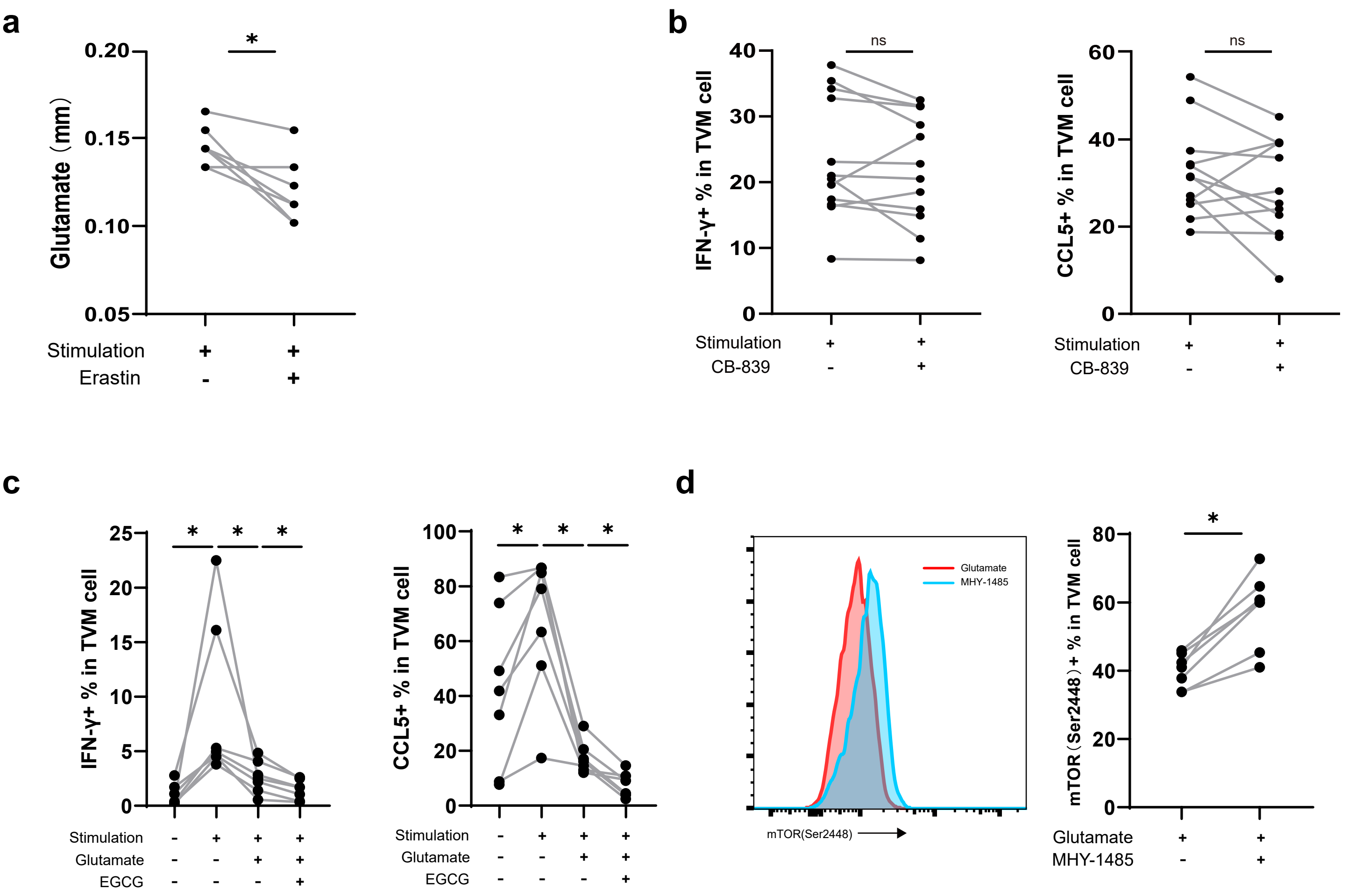

Supplementary Figure 9. The impacts of glutamate metabolism on the anti-HIV function of TVM cells. **a** The effect of Erastin intervention on glutamate concentration in the culture medium. **b** The anti-HIV function of TVM cells upon treatment with glutamine catabolism inhibitors (Telaglenastat, CB-839). **c** The anti-HIV function of TVM cells upon treatment with glutamate catabolism inhibitors (Epigallocatechin Gallate, EGCG). **d** Alterations in mTORC1 pathway activity after mTORC1 activator (MHY-1485) intervention. *P < 0.05

Supplement Table 1. Clinical characteristics of participants enrolled in plasma metabolites detections

characteristic	HC (n=11)	ART (n=59)
Age (y)	27(24-33)	46 (26–56)
Gender (female/male)	7/4	22/37
Viral load (Log10/ml)	-	< LOD
ART regimens		
2 NRTIs+ 1 NNRTIs	-	49
2 NRTIs+ 1 PIs	-	9
1 NRTIs+ 1PIs	-	1
Pre-ART		
CD4 Count (cells/μL)	-	172 (5-934)
CD8 Count (cells/μL)	-	589 (50-2650)
CD4/CD8 ratio	-	0.23 (0.01-1.23)
At enrollment		
CD4 Count (cells/μL)	-	517 (283–1378)
CD8 Count (cells/μL)	-	752 (377–1846）
CD4/CD8 ratio	-	0.70 (0.24–1.87）

Data are shown as medians with ranges (min to max). ART, Antiretroviral therapy; HC, Health control; LOD, limit of detection. NRTIs, nucleoside reverse transcriptase inhibitors; NNRTIs, non-nucleoside reverse transcriptase inhibitors; PIs, protease inhibitors.

Supplement Table 2. Comparison of glutamate levels between different clinical groups

Characteristic			P Value
Gender	Male	Female	
38/21	(1131.33,1558.95)	-8,751,357.20	0.02
ART regimens	2 NRTIs + 1 NNRTIs	Others	
49/10	(975.25,1498.05)	-8,751,232.80	0.295
ART duration	＜5 years	＞5 years	
20/39	(897.9,1369.8)	(951.1,1481.5)	0.305
CD4 count (cells/ul)	＞500	＜500	
30/29	(975.3,1449.8)	(895.3,1524.7)	0.83
CD8 count（cells/ul）	＞1000	＜1000	
17/42	（1126.3,1572.9）	（873.8,1444.75）	0.21
CD4/CD8	＞1	＜1	
12/47	(890.2,1430.7)	(981.6,1484.1)	0.68

Data are shown as interquartile range. ART, Antiretroviral therapy; NRTIs, nucleoside reverse transcriptase inhibitors; NNRTIs, non-nucleoside reverse transcriptase inhibitors.

Supplement Table 3. Signature genes usded to calculate of functional scores

cytotoxic	inflammatory	effector	proliferation	glutamate transmembrane transport	glutamate catabolic process to aspartate	aspartate metabolic process	asparagine metabolic process
PRF1	IRF1	GZMA	ADRA1D	ATP1A2	GOT1	ADSS2	ASNS
IFNG	CD8A	GZMB	CCKBR	CLN8	GOT2	ASPA	ATF4
GNLY	CCL2	PRF1	CD3E	GRM1		ASS1	ASNSD1
NKG7	CCL3	EOMES	CD86	ITGB1		GOT1	NIT2
GZMB	CCL4	IFNG	CDK2	KCNJ10		GOT2	ASRGL1
GZMA	CXCL9	TNF	CIAO1	NTSR1		DDO	
GZMH	CXCL10	CXCL9	CSF1	PSEN1		ADSS1	
KLRK1	ICOS	CXCL10	CSF3	SLC1A1		GOT1L1	
KLRB1	GZMK	CD8A	CTF1	SLC1A2			
KLRD1	HLA-DMA	CD4	CXCL5	SLC1A3			
CTSW	HLA-DMB	FOXP3	DDX11	SLC1A6			
CST7	HLA-DOA	ICOS	DHPS	SLC1A7			
	HLA-DOB	CTLA4	EDN1	SLC3A1			
			EGF	SLC25A12			
			FGB	PER2			
			FGF7	SLC25A13			
			FGG	ARL6IP5			
			FLT1	PRAF2			
			FOSL1	ARL6IP1			
			GRN	SLC7A11			
			HBEGF	SLC38A7			
			IGF1	SLC17A7			
			IL3	SLC17A6			
			IL6	SLC25A22			
			LIF	SLC25A18			
			NAMPT	SLC7A13			
			NRP1	SLC17A8			
			PTN				
			S1PR2				
			SLAMF1				
			SSR1				
			STIM1				
			TIMP1				
			TNFSF4				
			VEGFC				
			VIPR1				

Supplement Table 4. Mass spectrum Multiple Reaction Monitoring acquisition parameters

Q1	Q3	RT	metabolites	DP	CE	CXP
193.8	136.8	1.77	Acatate	-109	-24	-8
207.8	136.9	2.43	Propionate	-122	-27	-8
221.9	151.9	3.31	Butyrate	-120	-20	-8
221.7	137	3.25	Isobutyrate	-122	-27	-4
236	137	4.24	Isovalerate	-147	-28	-16
550.3	233	7.61	α-ketoglutaric acid	-120	-37	-7
357.3	137.1	6.72	Pyruvic acid	-116	-30	-8
148	102	100	Glutamate	72	15	6
176	130	100	Indoleacetic acid	145	20	10
133.6	106.1	100	Oxindole	120	27	6
495.2	175	2.52	Fucose	115	25	11
552.2	175	2.45	GlcNAc	97	30	11

RT, retention time; DP, declustering potential; CE, Collision Energy; CXP, cell exit potential.
