# Supplementary material for: Elevated glutamate impedes anti-HIV-1 CD8 + T cell responses in HIV-1-infected individuals on antiretroviral therapy
Source: Commun Biol. 2023 Jul 7;6:696. doi: 10.1038/s42003-023-04975-z (PMC10328948; doi:10.1038/s42003-023-04975-z)
Supplement: Supplementary file 3 — Description of Additional Supplementary Files [file 42003_2023_4975_MOESM3_ESM.pdf]

Description of Additional Supplementary Files

File name: Supplementary Data 1

Description: The source data underlying the graphs and charts presented in the main figures.
