# Supplementary material for: Elevated glutamate impedes anti-HIV-1 CD8 + T cell responses in HIV-1-infected individuals on antiretroviral therapy
Source: Commun Biol. 2023 Jul 7;6:696. doi: 10.1038/s42003-023-04975-z (PMC10328948; doi:10.1038/s42003-023-04975-z)
Supplement: Supplementary file 5 — Reporting Summary [file 42003_2023_4975_MOESM5_ESM.pdf]

nature portfolio

Corresponding author(s): Fu-Sheng Wang

Last updated by author(s): May 12, 2023

Reporting Summary

Nature Portfolio wishes to improve the reproducibility of the work that we publish. This form provides structure for consistency and transparency in reporting. For further information on Nature Portfolio policies, see our Editorial Policies and the Editorial Policy Checklist.

Statistics

For all statistical analyses, confirm that the following items are present in the figure legend, table legend, main text, or Methods section.

n/a	Confirmed	
☐	☒	The exact sample size (*n*) for each experimental group/condition, given as a discrete number and unit of measurement
☐	☒	A statement on whether measurements were taken from distinct samples or whether the same sample was measured repeatedly
☐	☒	The statistical test(s) used AND whether they are one- or two-sided<br>*Only common tests should be described solely by name; describe more complex techniques in the Methods section.*
☐	☒	A description of all covariates tested
☐	☒	A description of any assumptions or corrections, such as tests of normality and adjustment for multiple comparisons
☐	☒	A full description of the statistical parameters including central tendency (e.g. means) or other basic estimates (e.g. regression coefficient) AND variation (e.g. standard deviation) or associated estimates of uncertainty (e.g. confidence intervals)
☐	☒	For null hypothesis testing, the test statistic (e.g. *F*, *t*, *r*) with confidence intervals, effect sizes, degrees of freedom and *P* value noted<br>*Give P values as exact values whenever suitable.*
☒	☐	For Bayesian analysis, information on the choice of priors and Markov chain Monte Carlo settings
☒	☐	For hierarchical and complex designs, identification of the appropriate level for tests and full reporting of outcomes
☐	☒	Estimates of effect sizes (e.g. Cohen's *d*, Pearson's *r*), indicating how they were calculated

Our web collection on statistics for biologists contains articles on many of the points above.

Software and code

Policy information about availability of computer code

Data collection	Main source data used to conduct scRNA-seq analysis can be accessed under the accession number http://bigd. big. ac. cn/gsa-human, accession code HRA000190.
Data analysis	FlowJo_v10, GraphPad Prism 8, RStudio, Python

For manuscripts utilizing custom algorithms or software that are central to the research but not yet described in published literature, software must be made available to editors and reviewers. We strongly encourage code deposition in a community repository (e.g. GitHub). See the Nature Portfolio guidelines for submitting code & software for further information.

Data

Policy information about availability of data

All manuscripts must include a data availability statement. This statement should provide the following information, where applicable:

- Accession codes, unique identifiers, or web links for publicly available datasets
- A description of any restrictions on data availability
- For clinical datasets or third party data, please ensure that the statement adheres to our policy

scRNA-seq data
The authors declare that the data supporting the findings of this study are available within the article, its Supplementary Information file, and upon reasonable request.

Human research participants

Policy information about studies involving human research participants and Sex and Gender in Research.

Reporting on sex and gender	Age, gender, clinical parameters like (CD4, CD8, viral load), treatment duration are detailed in the study
Population characteristics	population characteristics are detailed in the study
Recruitment	N/A
Ethics oversight	The study was approved by the the Ethics Committee of the Fifth Medical Center of Chinese PLA General Hospital 2016164D.

Note that full information on the approval of the study protocol must also be provided in the manuscript.

Field-specific reporting

Please select the one below that is the best fit for your research. If you are not sure, read the appropriate sections before making your selection.

☒ Life sciences ☐ Behavioural & social sciences ☐ Ecological, evolutionary & environmental sciences

For a reference copy of the document with all sections, see nature.com/documents/nr-reporting-summary-flat.pdf

Life sciences study design

All studies must disclose on these points even when the disclosure is negative.

Sample size	Single cell RNA-seq data from the PBMC of 5 treatment-naïve (TN) PLWH, 3 ART-treated individuals and 4 healthy donors were were reanalyzed from an existing study published by Wang et al. 11 healthy donors, 59 ART-treated individuals were included for liquid chromatography-mass spectrometry (LC-MS) analysis.
Data exclusions	N/A
Replication	In addition, given the limited amount of blood received for patient per analysis and the high degree of lymphopenia amongst the samples, itwas not possible to perform every experiment with all the patients in our cohort.
Randomization	Not relevant
Blinding	The investigator was not blinded as analysis on patient character stics and biological readout were conducted in the same laboratory.

Reporting for specific materials, systems and methods

We require information from authors about some types of materials, experimental systems and methods used in many studies. Here, indicate whether each material, system or method listed is relevant to your study. If you are not sure if a list item applies to your research, read the appropriate section before selecting a response.

Materials & experimental systems

n/a	Involved in the study	
☐	☒	Antibodies
☒	☐	Eukaryotic cell lines
☒	☐	Palaeontology and archaeology
☒	☐	Animals and other organisms
☐	☒	Clinical data
☒	☐	Dual use research of concern

Methods

n/a	Involved in the study	
☒	☐	ChIP-seq
☐	☒	Flow cytometry
☒	☐	MRI-based neuroimaging

Antibodies

Antibodies used	Are provided in the manuscript
Validation	All antibodies used in the manuscript are well-established by manufactures and other publications

Clinical data

Policy information about clinical studies

All manuscripts should comply with the ICMJE guidelines for publication of clinical research and a completed CONSORT checklist must be included with all submissions.

Clinical trial registration	N/A
Study protocol	N/A
Data collection	At the Fifth Medical Center of Chinese PLA General Hospital and the Fourth People's Hospital of Nanning
Outcomes	The correlation between plasma metabolites levels and HIV-1 reservoir size and CD8+ T cell function

Flow Cytometry

Plots

Confirm that:

☒ The axis labels state the marker and fluorochrome used (e.g. CD4-FITC).

☒ The axis scales are clearly visible. Include numbers along axes only for bottom left plot of group (a 'group' is an analysis of identical markers).

☒ All plots are contour plots with outliers or pseudocolor plots.

☒ A numerical value for number of cells or percentage (with statistics) is provided.

Methodology

Sample preparation	PBMCs were resuspended and incubated in RPMI 1640 containing 10% fetal bovine serum (FBS) at 37 °C and 5% CO_2 for 2 h. CD8+ T and TVM cell functions were detected as described in our previous study 24. Briefly, surface markers were stained with monoclonal antibodies (mAbs) for 30 min at 4 °C. For intracellular staining, samples were fixed and permeabilized using a Foxp3/transcription factor staining buffer set (Thermo Fisher Scientific Inc., Waltham, MA) and stained intracellularly with the indicated antibodies for 30 min at 4 °C. The stained samples were examined by flow cytometry on a BD Canto II flow cytometer (BD Biosciences, San Diego, CA, USA). Flow cytometry data were analyzed using FlowJo software (version 10.5.3; BD Biosciences).
Instrument	BD Canto II flow cytometer
Software	BD FACSDiva
Cell population abundance	N/A
Gating strategy	Provided in the supplementary materials

☒ Tick this box to confirm that a figure exemplifying the gating strategy is provided in the Supplementary Information.
